# Supplementary material for: Fish-hunting cone snail disrupts prey’s glucose homeostasis with weaponized mimetics of somatostatin and insulin
Source: Nat Commun. 2024 Aug 20;15:6408. doi: 10.1038/s41467-024-50470-2 (PMC11336141; doi:10.1038/s41467-024-50470-2)
Supplement: Supplementary file 1 — Supplementary Information [file 41467_2024_50470_MOESM1_ESM.docx]

**Supporting Information for**

**Fish-hunting cone snail disrupts prey's glucose homeostasis with weaponized mimetics of somatostatin and insulin**

Ho Yan Yeung^1,2^, Iris Bea L. Ramiro^1^, Daniel B. Andersen^1,3^, Thomas Lund Koch^1,2,4^, Alexander Hamilton^5,6^, Walden E. Bjørn-Yoshimoto^1^, Samuel Espino^4^, Sergey Y. Vakhrushev^7^, Kasper B. Pedersen^7^, Noortje de Haan^8^, Agnes L. Hipgrave Ederveen^8^, Baldomero M. Olivera^4^, Jakob G. Knudsen^5^, Hans Bräuner-Osborne^9^, Katrine T. Schjoldager^7^, Jens Juul Holst^1,3^, Helena Safavi-Hemami^1,2,4*^

^1^Department of Biomedical Sciences, University of Copenhagen, Blegdamsvej 3, DK-2200 Copenhagen N, Denmark.

^2^Department of Biochemistry, University of Utah, 15 N Medical Drive, Salt Lake City, UT 84112, USA.

^3^Novo Nordisk Foundation Centre for Basic Metabolic Research, Blegdamsvej 3, DK-2200 Copenhagen N, Denmark.

^4^School of Biological Sciences, University of Utah, 257 South 1400 East, Salt Lake City, UT 84112, USA.

^5^Department of Biology, University of Copenhagen, Ole Maaløes Vej 5, DK-2200 Copenhagen N, Denmark.

^6^Department of Clinical Sciences in Malmö, Islet Cell Exocytosis, Lund University, Malmö, Sweden.

^7^Copenhagen Center for Glycomics, Department of Cellular and Molecular Medicine, University of Copenhagen, Blegdamsvej 3, DK-2200 Copenhagen N, Denmark.

^8^Leiden University Medical Center, Center for Proteomics and Metabolomics, 2333 ZA Leiden, The Netherlands.

^9^Department of Drug Design and Pharmacology, University of Copenhagen, Jagtvej 160, DK-2100 Copenhagen, Denmark.

*Corresponding author: Helena Safavi-Hemami, Department of Biochemistry, University of Utah, 15 N Medical Drive, Salt Lake City, UT 84112, USA, email: helena.safavi@utah.edu

**This file includes:**

Figures S1 to S16

Tables S1 to S9

Supporting Information


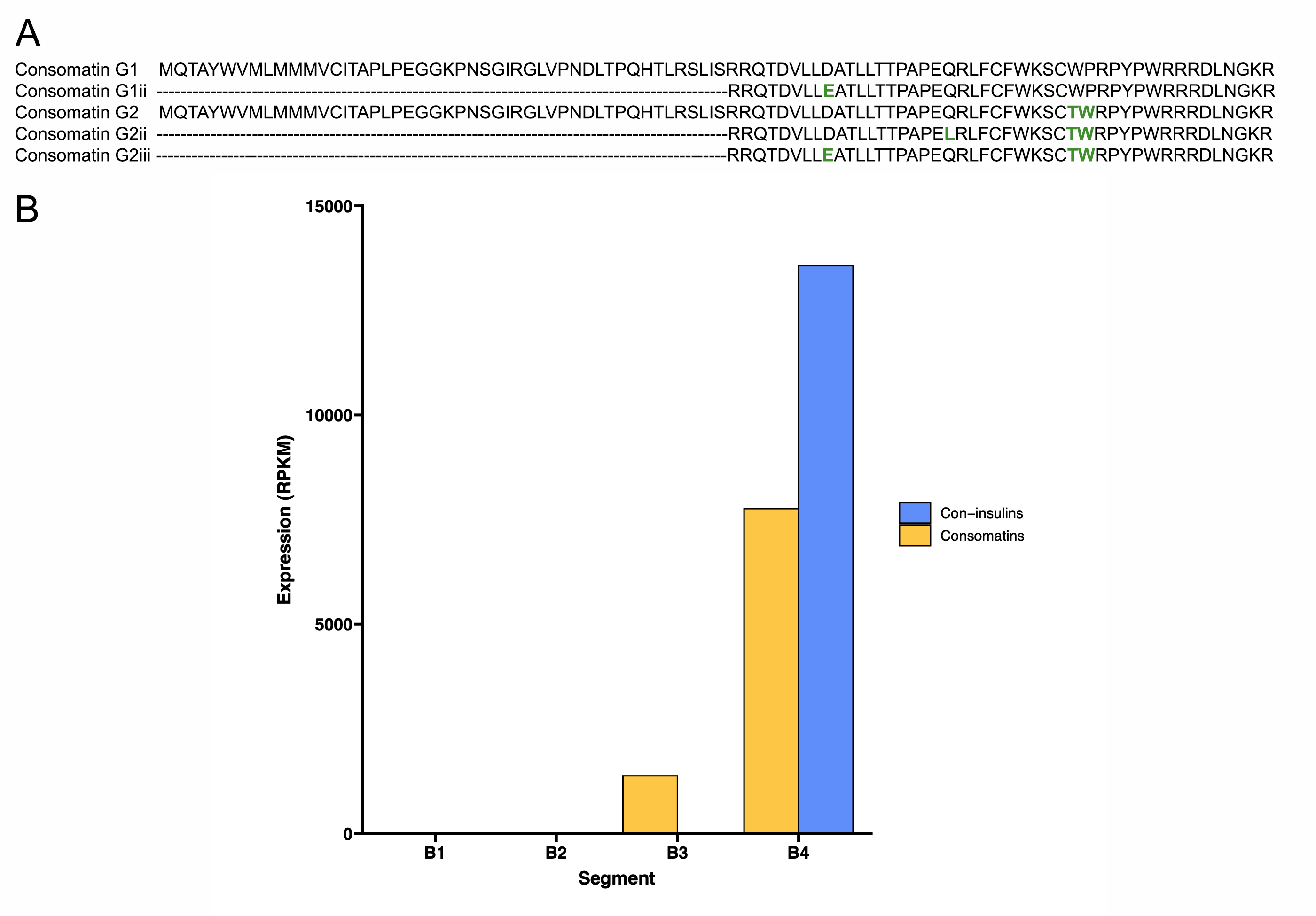


**Fig. S1. A)** Alignment of consomatin sequences retrieved from the venom gland transcriptomes of *C. geographus*. Names of sequences ending on ii or iii represent allelic variants retrieved by remapping of raw reads. Amino acids that differ from Consomatin G1 are shown in green. **B)** Differential expression of con-insulins and consomatins across *C. geographus* venom duct segments based on 454 RNAseq. Segment B1 is the most proximal and B4 most distal. RNAseq data (SRR403413- SRR403416) was accessed from the NCBI Sequence Read Archieve. Differential expression of identified con-insulins and consomatin transcripts across the venom gland was investigated by mapping all reads to the transcripts using Bowtie2 (--local --score-min G,60,6) and quantified using samtools (sort and idxstats) to calculate RPKM values.

Fig. S2. pG1 exhibits selective activation of hSSTR_2_. A to E) Representative dose-response curves of consomatin pG1 when tested at five different human SSTRs (SSTR_1_ - SSTR_5_) in comparison to octreotide and SS-14 using PRESTO-Tango β-arrestin recruitment assay. HTLA cell lines transiently transfected with five different human “Tango-ized” SSTRs were used in the PRESTO-Tango β-arrestin recruitment assay. All data were normalized to the SS-14 concentration-response curves at the respective hSSTRs. Error bars represent means $\boldsymbol{\pm}$ S.E.M. Experiments were conducted in triplicate, n = 4. Detailed pharmacological parameters of the dose response curves are further described in Table S1. F) Scattered plot illustrating the differences in potencies (pEC_50_) of SS-14, octreotide and pG1-induced SSTRs activation using PRESTO-Tango β-arrestin recruitment assay. pEC_50_ values for octreotide and pG1 at SSTR_1_, SSTR_3_, SSTR_4_ and SSTR_5_ were not included due to weak activation at the receptors (indicated by X). Pharmacological parameters of individual SSTR activation by SS-14, octreotide and pG1 are detailed in Table S1.

Fig. S3. Consomatin pG1 shows weaker potencies in dissociating different Gα_i/o_ protein compared to SS-14 and octreotide. A to E) Scattered plots illustrating the differences in potencies (pEC_50_) of SS-14, octreotide and pG1-induced Gα_i/o_ protein dissocation at the hSSTR_2_. Gα_i/o_ protein dissocation were measured using methods described by Masuho et al.^1^ in HEK293T cells transiently transfected with 3xHA-tagged human SSTR_2_, untagged Gα_i/o_ protein, Venus 156-239-Gβ, Venus 1-155-Gγ and masGRK3ct-Nluc. Error bars represent means $\boldsymbol{\pm}$ S.E.M. of at least three technical replicates. Detailed pharmacological parameters of the dose response curves are further described in Table S2. Statistical significance compared between individual peptide ligand responses in mediating different G protein dissocation were determined by one-way ANOVA with Turkey multiple comparison test (*, p < 0.05 (p = 0.0141 for Gi1; p = 0.0103 for GoA); ***, p = 0.0002; ns, non-statistically significant). Pharmacological parameters of Gi1, Gi2, Gi3, GoA and GoB activation at the SSTR_2_ by SS-14, octreotide and pG1 are detailed in Table S2.

**Fig. S4.** pG1 did not affect insulin and somatostatin output at low glucose (3.5 mM) in rat pancreas perfusion model. **A and B)** 0.1 nM, 1 nM and 10 nM pG1 were administered at three different time period throughout 100 mins measurement. 15 mins washout period were performed between the administration of different concentrations of pG1. 1 0mM L-arginine was applied for 10 mins towards the end of the experiment. Data represents means $\pm$ S.E.M. measured for n = 5 rats for measuring insulin output and n = 10 rats for measuring somatostatin output. **C and D)** Scattered plots illustrate the comparison of average glucagon output (fmol/min) of different doses of pG1 with baseline. Data represents means $\pm$ S.E.M. measured for n = 5 - 10 rats. n.s. means p > 0.05 (one-way repeated measure ANOVA - Dunnett test).

Fig. S5. pG1 to activate Dr-sstr2a but not Dr-sstr2b in G protein dissociation assay. A and B) Representative dose-response curve of consomatin pG1 when tested at Dr-sstr2a and Dr-sstr2b in comparison to SS-14 using G protein dissociation assay. HEK293T cell lines transiently transfected with 3xHA-tagged zebrafish Dr-sstr2a and Dr-sstr2b together with untagged Gα_i/o_ protein, Venus 156-239-Gβ, Venus 1-155-Gγ and masGRK3ct-Nluc were used in the G protein dissociation assay. All data were normalized to the SS-14 concentration-response curves. Error bars represent means $\boldsymbol{\pm}$ S.E.M. of n = 3 in technical duplicates. Pharmacological parameters of GoA dissociation at the Dr-sstr2a and Dr-sstr2b induced by SS-14 and pG1 are detailed in Table S4.

Fig. S6. Pool 6 and 7 of *C. geographus* venom fractions showed potent responses at mediating G_oA_ dissociation at the hSSTR_2_. Crude *C. geographus* venom was subjected to reversed-phase high performance liquid chromatography (RP-HPLC). Pools consisting of 5 adjacent fractions were then tested for their ability to induce G_oA_ dissociation at the hSSTR_2_. Pool 6 and 7 (corresponding to individual fraction 26-35) showed potent dose-dependent responses at inducing G_oA_ dissociation at A) hSSTR_2_, while having no effect at B) mock (pcDNA) transfected HEK293T cells. GoA protein dissocation were measured using methods described by Masuho et al.^1^ in HEK293T cells transiently transfected with 3xHA-tagged human SSTR_2_, untagged Gα_oA_ protein, Venus 156-239-Gβ, Venus 1-155-Gγ and masGRK3ct-Nluc. Error bars represent means $\boldsymbol{\pm}$ S.E.M. of only one technical experiment in duplicate.

Fig. S7. *C. geographus* venom fraction 32 (at 1:100 dilution) showed the most potent response at inducing G_oA_ dissocation at the hSSTR_2_. A) Fraction 26 to 36 show dose-dependent effect of mediating GoA dissocation at the hSSTR_2_. Fraction 32 in particular show potent response of G_oA_ dissociation despite the fraction was diluted by one-hundred fold, and was therefore selected for further MS/MS identification of the native G1. B) Dose-dependent response of G_oA_ dissocation was absent in mock (pcDNA) transfected HEK293T cells. G_oA_ protein dissocation were measured using methods described by Masuho et al.^1^ in HEK293T cells transiently transfected with 3xHA-tagged human SSTR_2_, untagged Gα_oA_ protein, Venus 156-239-Gβ, Venus 1-155-Gγ and masGRK3ct-Nluc. Error bars represent means $\boldsymbol{\pm}$ S.E.M. of only one technical experiment in duplicate.


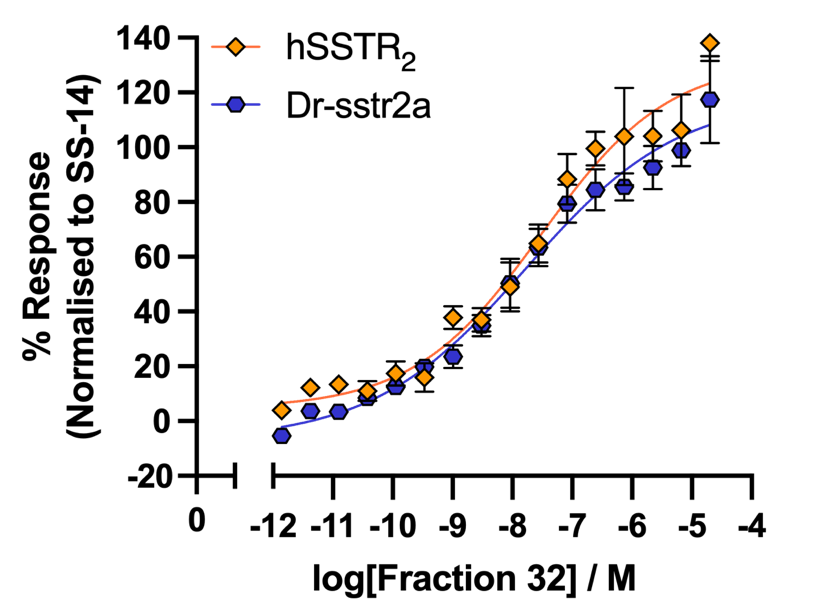


Fig. S8. Fraction 32 is equipotent at activating both hSSTR_2_ and Dr-sstr2a. Fraction 32 activated both hSSTR_2_ and Dr-sstr2a potently in PRESTO-Tango β-arrestin recruitment assay. Error bars represent means $\boldsymbol{\pm}$ S.E.M. of n = 2 in technical duplicates.

Fig. S9. Fraction # 32 subfraction screening at human SSTR_1_ to SSTR_5_, zebrafish Dr-sstr2a. A to E) Representative dose-response curves of different fraction # 32 subfractions when tested at five different human SSTR (SSTR_1_-SSTR_5_) using PRESTO-Tango β-arrestin recruitment assay. HTLA cell lines transiently transfected with five different human “Tango-ized” SSTRs and zebrafish Dr-sstr2a and Dr-sstr2b were used in the PRESTO-Tango β-arrestin recruitment assay. All data were normalized to the SS-14 concentration-response curves at the respective SSTRs. Error bars represent means $\boldsymbol{\pm}$ S.E.M. of three technical replicates in duplicates.

**Fig. S10.** Subfraction # 32-20 suppresses glucagon output at low glucose (3.5 mM) in rat pancreas perfusion model. **A)** 0.1 nM and 1 nM #32-20 were administered at two different time period throughout 90 mins measurement. 25 mins washout period were performed between the administration of two different concentrations of #32-20. 10mM L-arginine was applied for 5 mins towards the end of the experiment. Data represents means $\pm$ S.E.M. measured for n = 5 rats. **B)** Scattered plots illustrate the comparison of average glucagon output (fmol/min) of two doses of #32-20 with baseline. Data represents means $\pm$ S.E.M. measured for n = 5 rats. ** means p = 0.0021; n.s. means p > 0.05 (one-way repeated measure ANOVA - Dunnett test).

**A**


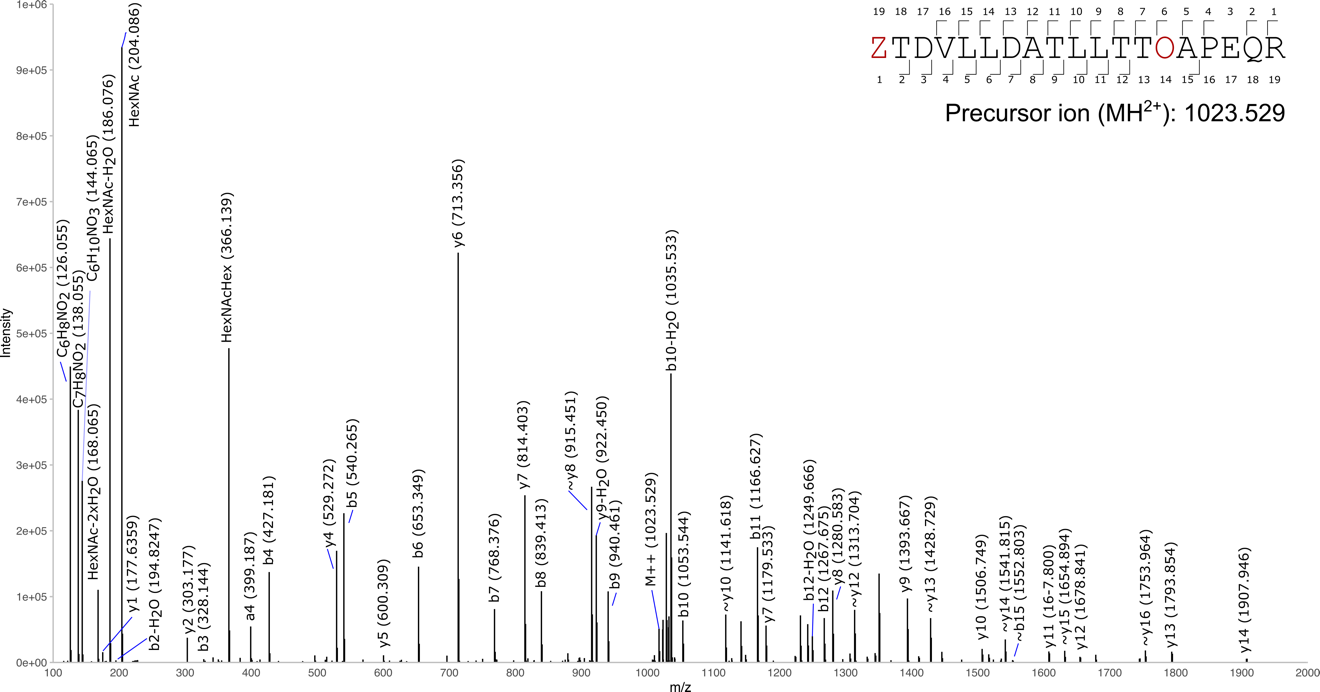


**B**


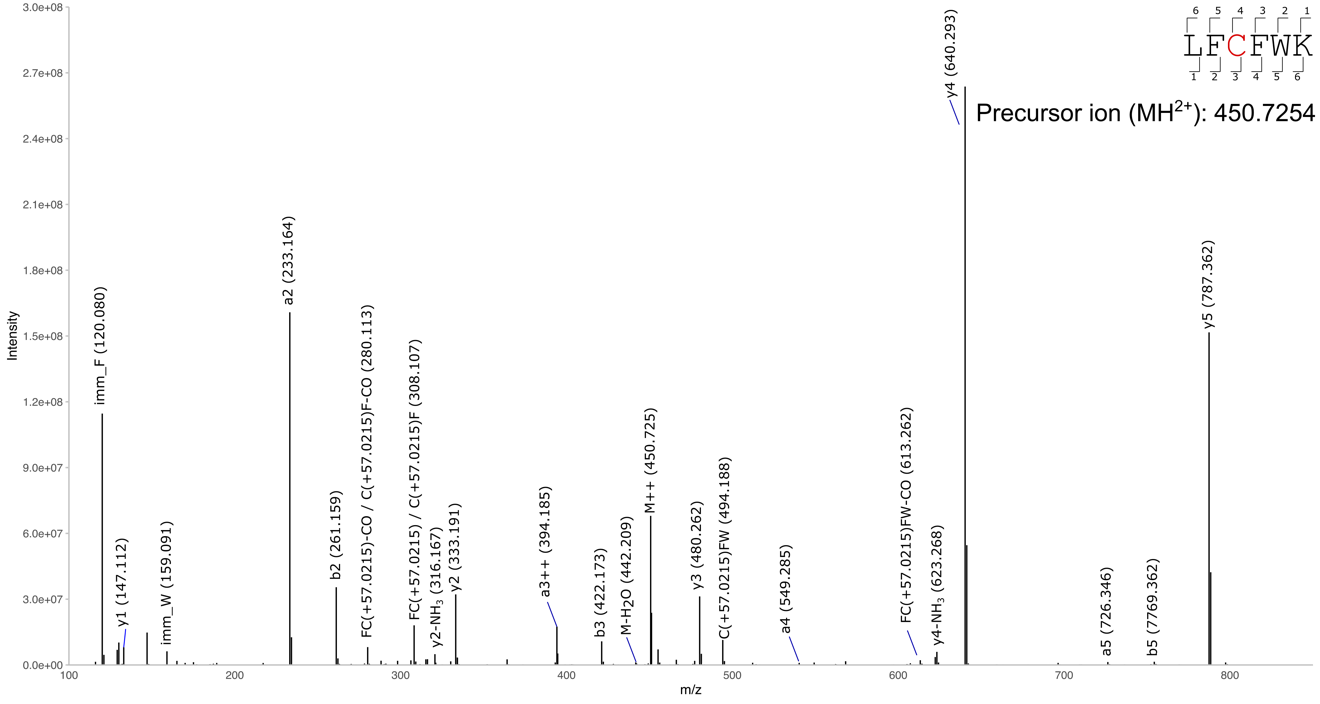


**C**

**
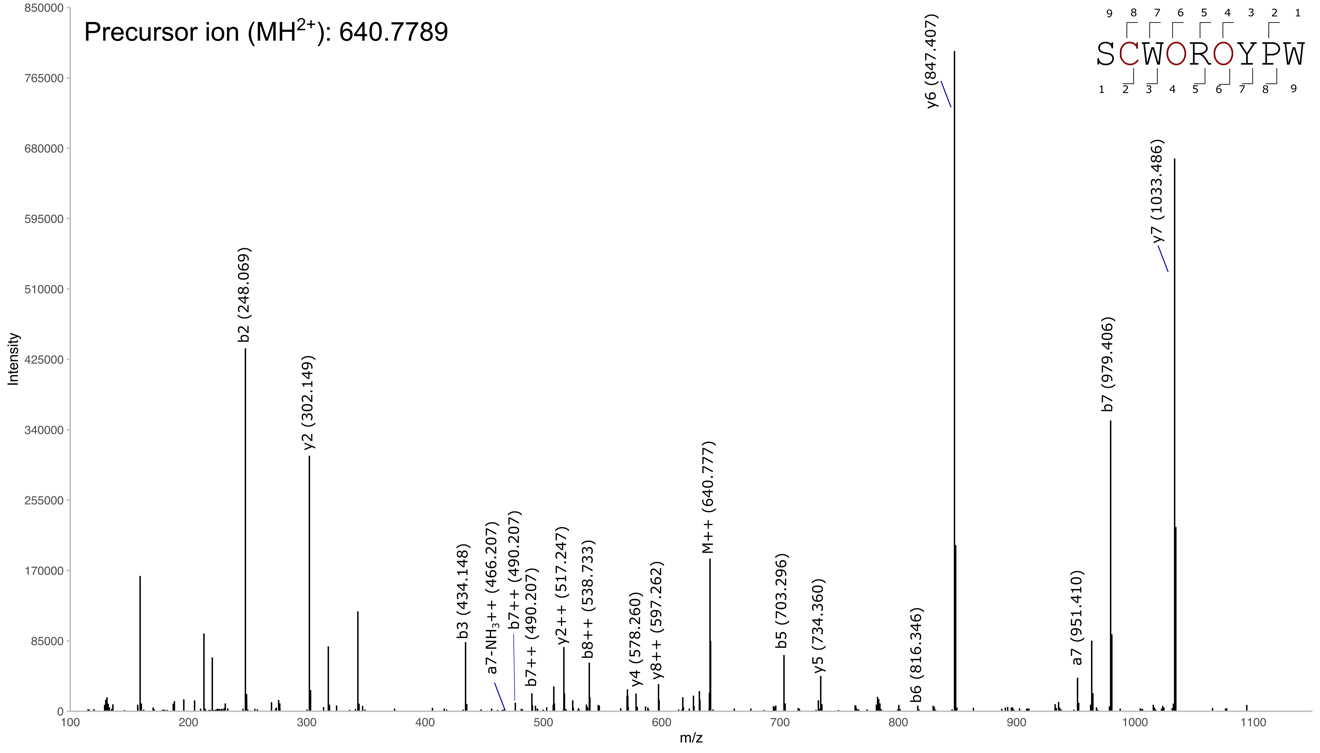
**

Fig. S11. Fragments of native consomatin G1 identified by tandem MS sequencing. Subfraction #32-20 was reduced, alkylated and trypsinized prior to interrogation by an Orbitrap mass spectrometer. Spectra were analysed using Byonic software (w2.16), and the results were manually inspected and annotated. A) shows the heavily glycosylated N-terminal tail yielding diagnostic HexNac and HexHexNac oxonium ions (m/z 204.08 and m/z 366.13, respectively) and various post-translational modifications (highlighted in red). B) shows the tryptic fragment of the consomatin G1 FWK motif with the cysteine undergoing derivatization (highlighted in red). C) shows the tryptic fragment of the C-terminal end of the native consomatin G1 displaying post-translational modifications (highlighted in red). Modifications: Z, pyroglutamic acid; O, hydroxyproline; C, derivatized-cysteine. N-terminal fragment ions (b) and C-terminal fragment ions (y) are indicated underneath and above the peptide sequence respsectively. 2+: doubly charged ions; imm: abbreviation of immonium ions. m/z of each b and y ion are annotated next to their corresponding ions. The mass spectrometry proteomics data have been deposited to the ProteomeXchange Consortium via the PRIDE [1] partner repository with the dataset identifier PXD052869 and 10.6019/PXD052869.

**A Consomatin nG1 variations:**

| Modifications | Sequence | Observed (MH+) | Calculated (MH+) | Delta Mass error (ppm) |
| --- | --- | --- | --- | --- |
| (Hex)_1_ (HexNAc)_1_+ (Hex)_1_ (HexNAc)_2_ (Deoxyhexose)_1_  / 3 hydroxyprolines | Q(-17.0265)TDVLLDATLLT(+365.1142)T(+714.2695)P(+15.9949)APEQRLFC(-1.0078)FWKSC(-1.0078)WP(+15.9949)RP(+15.9949)YPW-Amidated | 5186.3949 | 5186.3769 | 3.104287 |
| (HexNAc)_2_ + (Hex)_1_ (HexNAc)_2_ (Deoxyhexose)_1_  / 4 hydroxyprolines | Q(-17.0265)TDVLLDATLLT(+406.1588)T(+714.2695)P(+15.9949)AP(+15.9949)EQRLFC(-1.0078)FWKSC(-1.0078)WP(+15.9949)RP(+15.9949)YPW | 5244.3880 | 5244.4004 | 2.364427 |
| (Hex)_1_ (HexNAc)_1_ + (Hex)_1_ (HexNAc)_2_ (Deoxyhexose)_2_  / 3 hydroxyprolines | Q(-17.0265)TDVLLDATLLT(+365.1142)T(+860.3274)P(+15.9949)APEQRLFC(-1.0078)FWKSC(-1.0078)WP(+15.9949)RP(+15.9949)YPW-Amidated | 5332.4455 | 5332.4348 | 1.537759 |
| (Hex)_1_ (HexNAc)_1_ (Deoxyhexose)_1_ +  (Hex)_1_ (HexNAc)_2_ (Deoxyhexose)_1_  / 3 hydroxyprolines | Q(-17.0265)TDVLLDATLLT(+511.1901)T(+714.2695)P(+15.9949)APEQRLFC(-1.0078)FWKSC(-1.0078)WP(+15.9949)RP(+15.9949)YPW-Amidated | 5332.4455 | 5332.4528 | 1.368976 |
| (HexNAc)_2_ + (Hex)_1_ (HexNAc)_2_ (Deoxyhexose)_2_  / 4 hydroxyprolines | Q(-17.0265)TDVLLDATLLT(+406.1588)T(+  860.3274)P(+15.9949)APEQRLFC(-1.0078)FWKSC(-1.0078)WP(+15.9949)RP(+15.9949)YPW | 5390.4453 | 5390.4583 | 3.951427 |

**B Consomatin nG2 variations:**

| Modifications | Sequence | Observed (MH+) | Calculated (MH+) | Delta Mass error (ppm) |
| --- | --- | --- | --- | --- |
| (Hex)_1_ (HexNAc)_1_+ (Hex)_1_ (HexNAc)_2_ (Deoxyhexose)_1_  / 3 hydroxyprolines | Q(-17.0265)TDVLLDATLLT(+365.1142)T(+714.2695)P(+15.9949)APEQRLFC(-1.0078)FWKSC(-1.0078)**T**WRP(+15.9949)YP(+15.9949)W | 5191.3809 | 5191.3559 | 4.642332 |
| (Hex)_1_ (HexNAc)_1_ (Deoxyhexose)_1_ +  (Hex)_1_ (HexNAc)_2_ (Deoxyhexose)_1_  / 3 hydroxyprolines | Q(-17.0265)TDVLLDATLLT(+511.1901)T(+714.2695)P(+15.9949)APEQRLFC(-1.0078)FWKSC(-1.0078)TWRP(+15.9949)YP(+15.9949)W | 5337.4320 | 5337.4318 | 0.037471 |
| (Hex)_1_ (HexNAc)_1_ + (Hex)_1_ (HexNAc)_2_ (Deoxyhexose)_2_  / 3 hydroxyprolines | Q(-17.0265)TDVLLDATLLT(+365.1142)T(+860.3274)P(+15.9949)APEQRLFC(-1.0078)FWKSC(-1.0078)**T**WRP(+15.9949)YP(+15.9949)W | 5337.4320 | 5337.4138 | 3.409891 |

**Fig. S12.** Intact mass search for Consomatin nG1 and nG2. **A)** Consomatin nG1 and **B)** Consomatin nG2 with different glycosomes were found in the intact mass search and manually validated. MH+ (mono) were predicted using ProteinProspector (version 6.4.9) with the following mass shifts: Q(pyroglutamate): -17.0265; hydroxylation: +15.9949; cysteine reduction: -1.0078. The exact positions of the hydroxylation of proline, valine or lysine residues are not determined in this study.

**A Edman sequencing** **B Transcriptomic prediction**

| Cycle number | Amino acid | Position | Amino acid | Amino acid | Amino acid |
| --- | --- | --- | --- | --- | --- |
| 0 |  | 0 | Q | Q | Q |
| 1 | T | 1 | T | T | T |
| 2 | D | 2 | D | D | D |
| 3 | V | 3 | V | V | V |
| 4 | L | 4 | L | L | L |
| 5 | L | 5 | L | L | L |
| 6 | D | 6 | D | D | D |
| 7 | A | 7 | A | A | A |
| 8 | T | 8 | T | T | T |
| 9 | L | 9 | L | L | L |
| 10 | L | 10 | L | L | L |
| 11 | ? | 11 | T | T | T |
| 12 | ? | 12 | T | T | T |
| 13 | ?*, I, P | 13 | O | O | O |
| 14 | A | 14 | A | A | A |
| 15 | P | 15 | P | P | P |
| 16 | E | 16 | E | E | E |
| 17 | Q, L | 17 | Q | Q | L |
| 18 | R | 18 | R | R | R |
| 19 | L | 19 | L | L | L |
| 20 | F | 20 | F | F | F |
| 21 | ? | 21 | C | C | C |
| 22 | F | 22 | F | F | F |
| 23 | W | 23 | w | w | w |
| 24 | K | 24 | K | K | K |
| 25 | S | 25 | S | S | S |
| 26 | ?, L, K | 26 | C | C | C |
| 27 | W, T | 27 | W | T | T |
| 28 | ?*, P | 28 | O | W | W |
| 29 | R | 29 | R | R | R |
| 30 | ?*, R | 30 | O | O | O |
| 31 | Y | 31 | Y | Y | Y |
| 32 | P | 32 | P | O | O |
| 33 | ?, P? | 33 | W | W | W |
| 34 | ?, R? |  | **Consomatin nG1** | **Consomatin nG2** | **Consomatin nG2ii** |

**Fig. S13.** Edman sequencing results of subfraction #32-20. **A)** N-terminal deblocking of pyroglutamate (Z) was performed prior to Edman sequencing (Cycle 0). ? at cycles 11 and 12 indicate the blockage of signal, suggesting the presence of modified threonine glycosylation sites. ?* at cycle 13, 28 and 30 indicate the presence of unidentifiable residues, presumably hydroxylproline, which cannot be resolved by Edman sequencing. Cycle 17 indicates the presence of both glutamate and lysine residues, suggesting the presence of the N-terminal of Consomatin nG2ii. ? at cycles 21 and 26 indicate the blockage of signal due to the presence of an underivatized disulfide loop. Cycle 27 suggest the presence of the C-terminal of both Consomatin nG1 and nG2. Cycles that cannot be resolved are highlighted in red. The resolution after cycle 27 is low due to the differences in the C terminal ends of Consomatin nG1 and nG2 proteoforms. **B)** Transcriptomic predictions of Consomatin nG1, nG2 and nG2ii aligned with the Edman sequencing results. Residues that differ between the three proteoforms are highlighted in green.


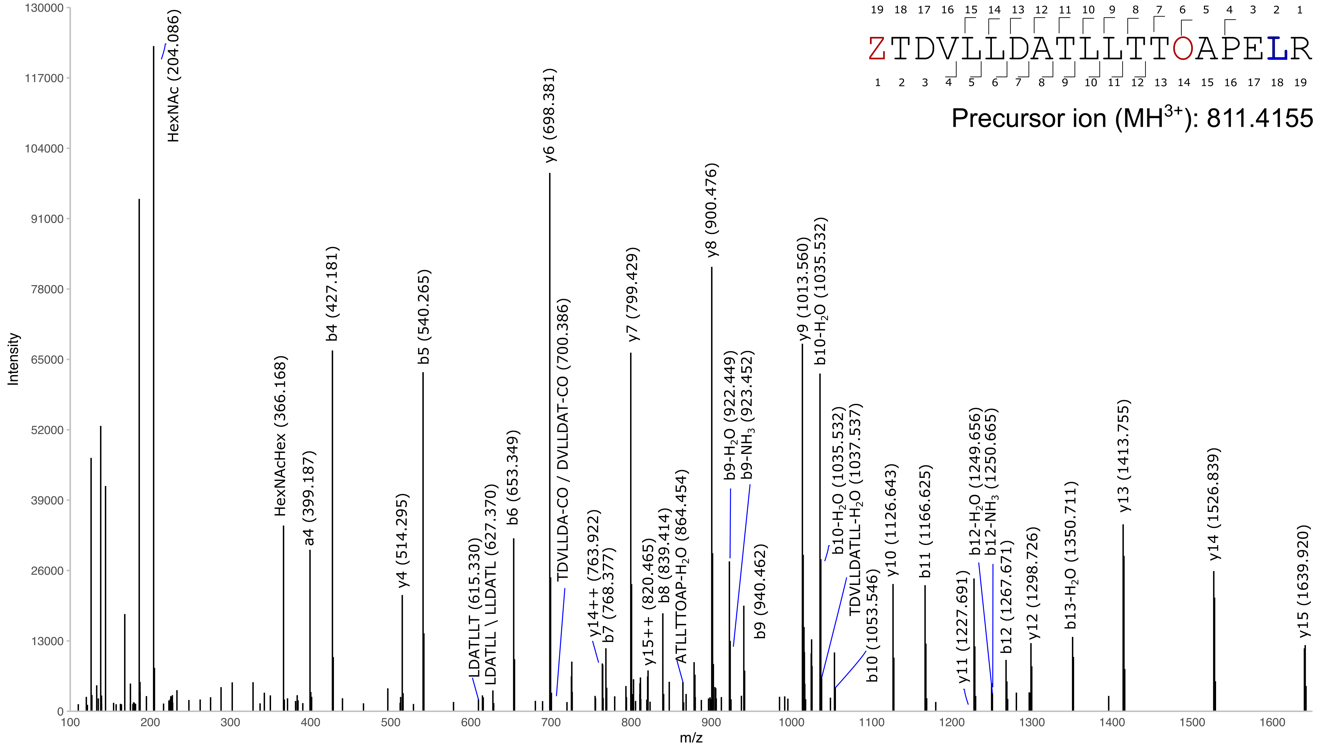


Fig. S14. Fragments of native consomatin G2ii identified by tandem MS sequencing. Subfraction # 32-20 was reduced, alkylated and trypsinized prior to interrogation by an Orbitrap mass spectrometer. Spectra were analysed using Byonic software (w2.16), and the results were manually inspected and annotated. The diagram shows the heavily glycosylated N-terminal tail yielding diagnostic HexNac and HexHexNac oxonium ions (m/z 204.08 and m/z 366.13, respectively) and various post-translational modifications (highlighted in red). Modifications: Z, pyroglutamic acid; O, hydroxyproline; C, derivatized-cysteine. N-terminal fragment ions (b) and C-terminal fragment ions (y) are indicated underneath and above the peptide sequence respsectively. 3+: triply charged ions; imm: abbreviation of immonium ions. m/z of each b and y ion are annotated next to their corresponding ions. The mass spectrometry proteomics data have been deposited to the ProteomeXchange Consortium via the PRIDE [1] partner repository with the dataset identifier PXD052869 and 10.6019/PXD052869.

Fig. S15. Des-glyco nG1 is a partial agonist at Dr-sstr2a. A to C) Representative dose-response curves of des-glyco nG1 when tested at human SSTR_2_, Dr-sstr2a and Dr-sstr2b in comparison to SS-14 and #32-20 using PRESTO-Tango β-arrestin recruitment assay. All data were normalized to the SS-14 concentration-response curves at the respective receptos. Error bars represent means $\boldsymbol{\pm}$ S.E.M. of n = 3 for SS-14, pG1 and des-glyco nG1 and n = 2 for #32-20 in technical duplicates. D) Scattered plot illustrating the differences in potencies (pEC_50_) of SS-14, #32-20 and des-glyco nG1-induced SSTRs activation using PRESTO-Tango β-arrestin recruitment assay. X indicates undeterminable pEC_50_ values due to receptor inactivation. Pharmacological parameters of individual SSTR activation by SS-14, #32-20 and des-glyco nG1 are detailed in Table S8.

Fig. S16. Des-glyco nG1 retains potent activation of hSSTR_2_. A to E) Representative dose-response curves of des-glyco nG1 when tested at five different human SSTRs (SSTR_1_ – SSTR_5_) in comparison to SS-14 and #32-20 using PRESTO-Tango β-arrestin recruitment assay. HTLA cell lines transiently transfected with five different human “Tango-ized” SSTRs were used in the PRESTO-Tango β-arrestin recruitment assay. All data were normalized to the SS-14 concentration-response curves at the respective hSSTRs. Error bars represent means $\boldsymbol{\pm}$ S.E.M. of n = 3 for SS-14 and des-glyco nG1 and n = 2 for #32-20 in technical duplicates. F) Scattered plot illustrating the differences in potencies (pEC_50_) of SS-14, #32-20 and des-glyco nG1-induced SSTRs activation using PRESTO-Tango β-arrestin recruitment assay. pEC_50_ values for #32-20 and des-glyco nG1 at SSTR_1_, SSTR_3_, SSTR_4_ and SSTR_5_ were not included (indicated by X) due to weak activation at the receptors.

Table S1. Pharmacological parameters of SS-14, octreotide and pG1 mediating SSTRs activation measured by PRESTO-Tango β-arrestin recruitment assay.

|  | Test ligands | **SS-14** | **Octreotide** | **pG1** |
| --- | --- | --- | --- | --- |
| **hSSTR_1_** | pEC_50_^a^ $\boldsymbol{\pm}$ S.E.M. | 8.22 $\boldsymbol{\pm}$ 0.41 | N.D. | N.D. |
|  | EC_50_ (nM) | 6.00 |  |  |
|  | E_max_^b^ $\boldsymbol{\pm}$ S.E.M. | 118.3 $\boldsymbol{\pm}$ 21.29 |  |  |
|  | Span $\boldsymbol{\pm}$S.E.M. | 140.34 $\boldsymbol{\pm}$ 47.20 |  |  |
|  | Hill Slope | 0.27 $\boldsymbol{\pm}$ 0.12 |  |  |
|  | n | 6 | 5 | 4 |
| **hSSTR_2_** | pEC_50_^a^ $\boldsymbol{\pm}$ S.E.M. | 7.73 $\boldsymbol{\pm}$ 0.04 | 9.05 $\boldsymbol{\pm}$ 0.04 | 8.45 $\boldsymbol{\pm}$ 0.06 |
|  | EC_50_ (nM) | 18.47 | 0.90 | 3.63 |
|  | E_max_^b^ $\boldsymbol{\pm}$ S.E.M. | 99.03 $\boldsymbol{\pm}$ 2.26 | 94.04 $\boldsymbol{\pm}$ 1.40 | 105.48 $\boldsymbol{\pm}$ 3.01 |
|  | Span $\boldsymbol{\pm}$S.E.M. | 97.00 $\boldsymbol{\pm}$ 2.97 | 96.38 $\boldsymbol{\pm}$ 3.43 | 106.03 $\boldsymbol{\pm}$ 6.98 |
|  | Hill Slope | 0.84 $\boldsymbol{\pm}$ 0.07 | 0.82 $\boldsymbol{\pm}$ 0.08 | 0.50 $\boldsymbol{\pm}$ 0.06 |
|  | n | 18 | 14 | 8 |
| **hSSTR_3_** | pEC_50_^a^ $\boldsymbol{\pm}$ S.E.M. | 7.02 $\boldsymbol{\pm}$ 0.12 | 5.85 | N.D. |
|  | EC_50_ (nM) | 9.64 | 1425.82 |  |
|  | E_max_^b^ $\boldsymbol{\pm}$ S.E.M. | 109.3 $\boldsymbol{\pm}$ 5.38 | 107.78 $\boldsymbol{\pm}$ 13.36 |  |
|  | Span $\boldsymbol{\pm}$S.E.M. | 110.18 $\boldsymbol{\pm}$ 6.27 | 108.83 $\boldsymbol{\pm}$ 13.66 |  |
|  | Hill Slope | 0.622 $\boldsymbol{\pm}$ 0.07 | 1.07 $\boldsymbol{\pm}$ 0.26 |  |
|  | n | 8 | 6 | 4 |
| **hSSTR_4_** | CEC_50_^a^ $\boldsymbol{\pm}$ S.E.M. | 8.66 $\boldsymbol{\pm}$ 0.17 | N.D. | N.D. |
|  | EC_50_ (nM) | 2.20 |  |  |
|  | E_max_^b^ $\boldsymbol{\pm}$ S.E.M. | 95.92 $\boldsymbol{\pm}$ 4.94 |  |  |
|  | Span $\boldsymbol{\pm}$S.E.M. | 79.41 $\boldsymbol{\pm}$ 8.37 |  |  |
|  | Hill Slope | 0.19 $\boldsymbol{\pm}$ 0.11 |  |  |
|  | n | 10 | 5 | 4 |
| **hSSTR_5_** | pEC_50_^a^ $\boldsymbol{\pm}$ S.E.M. | 7.63 $\boldsymbol{\pm}$ 0.04 | 6.56 $\boldsymbol{\pm}$ 0.11 | N.D. |
|  | EC_50_ (nM) | 23.26 | 273.29 |  |
|  | E_max_^b^ $\boldsymbol{\pm}$ S.E.M. | 98.10 $\boldsymbol{\pm}$ 1.83 | 87.04 $\boldsymbol{\pm}$ 4.84 |  |
|  | Span $\boldsymbol{\pm}$S.E.M. | 98.31 $\boldsymbol{\pm}$ 2.34 | 87.47 $\boldsymbol{\pm}$ 5.28 |  |
|  | Hill Slope | 0.77 $\boldsymbol{\pm}$ 0.05 | 1.40 $\boldsymbol{\pm}$ 0.25 |  |
|  | n | 9 | 8 | 4 |

Values were generated when the data were fitted to the four-parameter logistic equation. Means ± S.E.M of n individual result sets were shown.

^a^ Negative logarithm of agonist concentration when reaching half maximal response.

^b^ % of maximal response observed when stimulated with ligands relative to SS-14.

Table S2. Pharmacological parameters of SS-14, octreotide and pG1 mediated Gi/o protein recruitment at the hSSTR_2_.

| **G protein** | Test ligands | **SS-14** | **Octreotide** | **pG1** |
| --- | --- | --- | --- | --- |
| **Gα_i1_** | pEC_50_^a^ $\boldsymbol{\pm}$ S.E.M. | 9.41 $\boldsymbol{\pm}$ 0.16 | 8.83 $\boldsymbol{\pm}$ 0.49 | 8.19 $\boldsymbol{\pm}$ 0.30 |
|  | EC_50_ (nM) | 0.39 | 1.48 | 6.46 |
|  | E_max_^b^ $\boldsymbol{\pm}$ S.E.M. | 94.43 $\boldsymbol{\pm}$ 4.40 | 84.25 $\boldsymbol{\pm}$ 4.44 | 95.59 $\boldsymbol{\pm}$ 8.52 |
|  | Span $\boldsymbol{\pm}$S.E.M. | 86.02 $\boldsymbol{\pm}$ 6.05 | 76.31 $\boldsymbol{\pm}$ 5.35 | 87.89 $\boldsymbol{\pm}$ 11.1 |
|  | Hill Slope | 1.11 $\boldsymbol{\pm}$ 0.26 | 1.26 $\boldsymbol{\pm}$ 0.31 | 0.81 $\boldsymbol{\pm}$ 0.28 |
|  | n | 6 | 3 | 6 |
| **Gα_i2_** | pEC_50_^a^ $\boldsymbol{\pm}$ S.E.M. | 9.33 $\boldsymbol{\pm}$ 0.24 | 9.16 $\boldsymbol{\pm}$ 0.02 | 8.87 $\boldsymbol{\pm}$ 0.15 |
|  | EC_50_ (nM) | 0.47 | 0.69 | 1.35 |
|  | E_max_^b^ $\boldsymbol{\pm}$ S.E.M. | 99.45 $\boldsymbol{\pm}$ 4.91 | 63.96 $\boldsymbol{\pm}$ 2.97 | 91.03 $\boldsymbol{\pm}$ 4.60 |
|  | Span $\boldsymbol{\pm}$S.E.M. | 92.78 $\boldsymbol{\pm}$ 6.38 | 62.98 $\boldsymbol{\pm}$ 3.64 | 83.42 $\boldsymbol{\pm}$ 5.67 |
|  | Hill Slope | 0.89 $\boldsymbol{\pm}$ 0.19 | 1.47 $\boldsymbol{\pm}$ 0.37 | 1.11 $\boldsymbol{\pm}$ 0.22 |
|  | n | 3 | 3 | 5 |
| **Gα_i3_** | pEC_50_^a^ $\boldsymbol{\pm}$ S.E.M. | 9.29 $\boldsymbol{\pm}$ 0.27 | 8.70 $\boldsymbol{\pm}$ 0.19 | 8.13 $\boldsymbol{\pm}$ 0.26 |
|  | EC_50_ (nM) | 0.51 | 2.00 | 7.41 |
|  | E_max_^b^ $\boldsymbol{\pm}$ S.E.M. | 93.74 $\boldsymbol{\pm}$ 2.73 | 77.50 $\boldsymbol{\pm}$ 3.67 | 75.17 $\boldsymbol{\pm}$ 7.38 |
|  | Span $\boldsymbol{\pm}$S.E.M. | 88.01 $\boldsymbol{\pm}$ 3.59 | 76.15 $\boldsymbol{\pm}$ 4.31 | 75.57 $\boldsymbol{\pm}$ 8.97 |
|  | Hill Slope | 1.34 $\boldsymbol{\pm}$ 0.24 | 0.85 $\boldsymbol{\pm}$ 0.14 | 0.70 $\boldsymbol{\pm}$ 0.18 |
|  | n | 5 | 4 | 5 |
| **Gα_oA_** | pEC_50_^a^ $\boldsymbol{\pm}$ S.E.M. | 9.58 $\boldsymbol{\pm}$ 0.18 | 8.73 $\boldsymbol{\pm}$ 0.38 | 8.29 $\boldsymbol{\pm}$ 0.29 |
|  | EC_50_ (nM) | 0.26 | 1.86 | 5.13 |
|  | E_max_^b^ $\boldsymbol{\pm}$ S.E.M. | 95.83 $\boldsymbol{\pm}$ 2.93 | 96.23 $\boldsymbol{\pm}$ 4.77 | 97.87 $\boldsymbol{\pm}$ 4.11 |
|  | Span $\boldsymbol{\pm}$S.E.M. | 94.80 $\boldsymbol{\pm}$ 4.18 | 89.64 $\boldsymbol{\pm}$ 5.74 | 97.45 $\boldsymbol{\pm}$ 6.80 |
|  | Hill Slope | 1.04 $\boldsymbol{\pm}$ 0.17 | 1.28 $\boldsymbol{\pm}$ 0.31 | 1.04 $\boldsymbol{\pm}$ 0.25 |
|  | n | 6 | 4 | 6 |
| **Gα_oB_** | pEC_50_^a^ $\boldsymbol{\pm}$ S.E.M. | 9.37 $\boldsymbol{\pm}$ 0.16 | 8.50 $\boldsymbol{\pm}$ 0.06 | 8.08 $\boldsymbol{\pm}$ 0.43 |
|  | EC_50_ (nM) | 0.43 | 3.16 | 8.32 |
|  | E_max_^b^ $\boldsymbol{\pm}$ S.E.M. | 91.91 $\boldsymbol{\pm}$ 2.83 | 100.59 $\boldsymbol{\pm}$ 2.64 | 76.66 $\boldsymbol{\pm}$ 3.62 |
|  | Span $\boldsymbol{\pm}$S.E.M. | 76.50 $\boldsymbol{\pm}$ 3.74 | 95.19 $\boldsymbol{\pm}$ 3.10 | 76.77 $\boldsymbol{\pm}$ 4.97 |
|  | Hill Slope | 1.15 $\boldsymbol{\pm}$ 0.24 | 1.32 $\boldsymbol{\pm}$ 0.17 | 1.46 $\boldsymbol{\pm}$ 0.41 |
|  | n | 4 | 3 | 4 |

Values were generated when the data were fitted to the four-parameter logistic equation. Means ± S.E.M of n individual result sets were shown.

^a^ Negative logarithm of agonist concentration when reaching half maximal response.

^b^ % of maximal response observed when stimulated with ligands relative to SS-14.

Table S3. Bias factor of SS-14, octreotide and pG1 mediated Gi/o protein dissocation at the hSSTR_2_.

| **G protein** | Test ligands | **SS-14** | **Octreotide** | **pG1** |
| --- | --- | --- | --- | --- |
| **Gα_i1_** | LogR $\boldsymbol{\pm}$ S.E.M. | 9.40 $\boldsymbol{\pm}$ 0.19 | 9.40 $\boldsymbol{\pm}$ 0.22 | 7.42 $\boldsymbol{\pm}$ 0.20 |
|  | ∆LogR $\boldsymbol{\pm}$ S.E.M. | 0 $\boldsymbol{\pm}$ 0 | 0.0071 $\boldsymbol{\pm}$ 0.29 | -1.98 $\boldsymbol{\pm}$ 0.27 |
|  | RE | 1 | 1.02 | 0.01 |
|  | N | 4 | 2 | 4 |
| **Gα_i2_** | LogR $\boldsymbol{\pm}$ S.E.M. | 9.35 $\boldsymbol{\pm}$ 0.29 | 8.19 $\boldsymbol{\pm}$ 0.45 | 7.87 $\boldsymbol{\pm}$ 0.12 |
|  | ∆LogR $\boldsymbol{\pm}$ S.E.M. | 0 $\boldsymbol{\pm}$ 0 | -1.17 $\boldsymbol{\pm}$ 0.53 | -1.48 $\boldsymbol{\pm}$ 0.31 |
|  | RE | 1 | 0.068 | 0.033 |
|  | N | 5 | 3 | 5 |
| **Gα_i3_** | LogR $\boldsymbol{\pm}$ S.E.M. | 9.71 $\boldsymbol{\pm}$ 0.10 | 8.96 $\boldsymbol{\pm}$ 0.12 | 8.30 $\boldsymbol{\pm}$ 0.24 |
|  | ∆LogR $\boldsymbol{\pm}$ S.E.M. | 0 $\boldsymbol{\pm}$ 0 | -0.75 $\boldsymbol{\pm}$ 0.16 | -1.41 $\boldsymbol{\pm}$ 0.26 |
|  | RE | 1 | 0.18 | 0.039 |
|  | N | 3 | 2 | 3 |
| **Gα_oA_** | LogR $\boldsymbol{\pm}$ S.E.M. | 9.84 $\boldsymbol{\pm}$ 0.08 | 9.40 $\boldsymbol{\pm}$ 0.13 | 8.75 $\boldsymbol{\pm}$ 0.10 |
|  | ∆LogR $\boldsymbol{\pm}$ S.E.M. | 0 $\boldsymbol{\pm}$ 0 | -0.44 $\boldsymbol{\pm}$ 0.15 | -1.08 $\boldsymbol{\pm}$ 0.13 |
|  | RE | 1 | 0.36 | 0.082 |
|  | N | 4 | 2 | 2 |
| **Gα_oB_** | LogR $\boldsymbol{\pm}$ S.E.M. | 9.62 $\boldsymbol{\pm}$ 0.04 | 8.89 $\boldsymbol{\pm}$ 0.0026 | 8.50 $\boldsymbol{\pm}$ 0.13 |
|  | ∆LogR $\boldsymbol{\pm}$ S.E.M. | 0 $\boldsymbol{\pm}$ 0 | -0.73 $\boldsymbol{\pm}$ 0.042 | -1.12 $\boldsymbol{\pm}$ 0.14 |
|  | RE | 1 | 0.18 | 0.076 |
|  | N | 2 | 2 | 3 |

**Table S4.** Pharmacological parameters of SS-14 and pG1 mediated Dr-sstr2a and Dr-sstr2b activation measured by PRESTO-Tango β-arrestin recruitment assay

|  | Test ligands | **SS-14** | **pG1** |
| --- | --- | --- | --- |
| **Dr-sstr2a** | pEC_50_^a^ $\boldsymbol{\pm}$ S.E.M. | 7.54 $\boldsymbol{\pm}$ 0.09 | N.D. |
|  | EC_50_ (nM) | 28.65 |  |
|  | E_max_^b^ $\boldsymbol{\pm}$ S.E.M. | 101.00 $\boldsymbol{\pm}$ 4.48 |  |
|  | Span $\boldsymbol{\pm}$S.E.M. | 98.28 $\boldsymbol{\pm}$5.77 |  |
|  | Hill Slope | 0.83 $\boldsymbol{\pm}$ 0.13 |  |
|  | n | 15 | 13 |
| **Dr-sstr2b** | pEC_50_^a^ $\boldsymbol{\pm}$ S.E.M. | 6.88 $\boldsymbol{\pm}$ 0.17 | 7.60 $\boldsymbol{\pm}$ 0.08 |
|  | EC_50_ (nM) | 131.9 | 25.32 |
|  | E_max_^b^ $\boldsymbol{\pm}$ S.E.M. | 120.27 $\boldsymbol{\pm}$ 10.12 | 83.12 $\boldsymbol{\pm}$ 3.13 |
|  | Span $\boldsymbol{\pm}$S.E.M. | 119.45 $\boldsymbol{\pm}$ 11.43 | 82.46 $\boldsymbol{\pm}$ 4.04 |
|  | Hill Slope | 0.51 $\boldsymbol{\pm}$ 0.07 | 0.90 $\boldsymbol{\pm}$ 0.13 |
|  | n | 6 | 6 |

Table S5. Pharmacological parameters of SS-14, Octreotide and pG1 mediated G_oA_ protein dissocation at the Dr-sstr2a and Dr-sstr2b.

| **GoA** | Test ligands | **SS-14** | **pG1** |
| --- | --- | --- | --- |
| **Dr-sstr2a** | pEC_50_^a^ $\boldsymbol{\pm}$ S.E.M. | 8.82 $\boldsymbol{\pm}$ 0.37 | N.D. |
|  | EC_50_ (nM) | 1.51 |  |
|  | E_max_^b^ $\boldsymbol{\pm}$ S.E.M. | 119.74 $\boldsymbol{\pm}$ 33.49 |  |
|  | Span $\boldsymbol{\pm}$S.E.M. | 98.92 $\boldsymbol{\pm}$ 37.05 |  |
|  | Hill Slope | 0.51 $\boldsymbol{\pm}$ 0.28 |  |
|  | n | 4 | 8 |
| **Dr-sstr2b** | pEC_50_^a^ $\boldsymbol{\pm}$ S.E.M. | 9.77 $\boldsymbol{\pm}$ 0.38 | 7.95 $\boldsymbol{\pm}$ 0.19 |
|  | EC_50_ (nM) | 0.17 | 11.22 |
|  | E_max_^b^ $\boldsymbol{\pm}$ S.E.M. | 110.48 $\boldsymbol{\pm}$ 23.67 | 105.52 $\boldsymbol{\pm}$ 10.37 |
|  | Span $\boldsymbol{\pm}$S.E.M. | 102.66 $\boldsymbol{\pm}$ 28.47 | 100.43 $\boldsymbol{\pm}$ 11.98 |
|  | Hill Slope | 0.38 $\boldsymbol{\pm}$ 0.17 | 0.48 $\boldsymbol{\pm}$ 0.10 |
|  | n | 10 | 10 |

Values were generated when the data were fitted to the four-parameter logistic equation. Means ± S.E.M of n individual result sets were shown.

^a^ Negative logarithm of agonist concentration when reaching half maximal response.

^b^ % of maximal response observed when stimulated with ligands relative to SS-14.

Table S6. Pharmacological parameters of *C. geographus* crude venom induced-Dr-sstr2a activation measured by PRESTO-Tango β-arrestin recruitment assay.

|  | Test ligands | *C. geographus* **crude venom** |
| --- | --- | --- |
| **hSSTR_1_^c^** | EC_50_ (mg/ml) | N.D. |
|  | E_max_^b^ $\boldsymbol{\pm}$ S.E.M. |  |
|  | Span $\boldsymbol{\pm}$S.E.M. |  |
|  | Hill Slope |  |
|  | n | 6 |
| **hSSTR_2_** | EC_50_ (mg/ml) | 0.010 $\boldsymbol{\pm}$ 0.001 |
|  | E_max_^b^ $\boldsymbol{\pm}$ S.E.M. | 100.04 $\boldsymbol{\pm}$ 4.83 |
|  | Span $\boldsymbol{\pm}$S.E.M. | 100.42 $\boldsymbol{\pm}$ 5.34 |
|  | Hill Slope | 1.16 $\boldsymbol{\pm}$ 0.16 |
|  | n | 8 |
| **hSSTR_3_^c^** | EC_50_ (mg/ml) | N.D. |
|  | E_max_^b^ $\boldsymbol{\pm}$ S.E.M. |  |
|  | Span $\boldsymbol{\pm}$S.E.M. |  |
|  | Hill Slope |  |
|  | n | 6 |
| **hSSTR_4_^c^** | EC_50_ (mg/ml) | N.D. |
|  | E_max_^b^ $\boldsymbol{\pm}$ S.E.M. |  |
|  | Span $\boldsymbol{\pm}$S.E.M. |  |
|  | Hill Slope |  |
|  | n | 6 |
| **hSSTR_5_ ^c^** | EC_50_ (mg/ml) | N.D. |
|  | E_max_^b^ $\boldsymbol{\pm}$ S.E.M. |  |
|  | Span $\boldsymbol{\pm}$S.E.M. |  |
|  | Hill Slope |  |
|  | n | 6 |
| **Dr-sstr2a** | EC_50_ (mg/ml) | 0.007 $\boldsymbol{\pm}$ 0.001 |
|  | E_max_^b^ $\boldsymbol{\pm}$ S.E.M. | 100.50 $\boldsymbol{\pm}$ 4.03 |
|  | Span $\boldsymbol{\pm}$S.E.M. | 101.32 $\boldsymbol{\pm}$ 4.60 |
|  | Hill Slope | 1.04 $\boldsymbol{\pm}$ 0.12 |
|  | n | 8 |
| **Dr-sstr2b** | EC_50_ (mg/ml) | 0.004 $\boldsymbol{\pm}$ 0.001 |
|  | E_max_^b^ $\boldsymbol{\pm}$ S.E.M. | 103.14 $\boldsymbol{\pm}$ 3.84 |
|  | Span $\boldsymbol{\pm}$S.E.M. | 103.31 $\boldsymbol{\pm}$ 4.58 |
|  | Hill Slope | 1.14 $\boldsymbol{\pm}$ 0.16 |
|  | n | 6 |

Values were generated when the data were fitted to the four-parameter logistic equation. Means ± S.E.M of n individual result sets were shown.

^a^ Negative logarithm of agonist concentration when reaching half maximal response.

^b^ % of maximal response observed when stimulated with ligands relative to SS-14.

^c^ Pharmacological parameters cannot be determined due to weak or inactivation of SSTRs.

Table S7. Pharmacological parameters of hSSTR_2_ and Dr-sstr2a activation induced by Fraction 32.

| **Receptor** | Test ligands | **Fraction 32** |
| --- | --- | --- |
| **hSSTR_2_** | pEC_50_^a^ $\boldsymbol{\pm}$ S.E.M. | 7.52 $\boldsymbol{\pm}$ 0.27 |
|  | EC_50_ (nM) | 30.20 |
|  | E_max_^b^ $\boldsymbol{\pm}$ S.E.M. | 131.86 $\boldsymbol{\pm}$ 12.53 |
|  | Span $\boldsymbol{\pm}$S.E.M. | 127.40 $\boldsymbol{\pm}$ 18.30 |
|  | Hill Slope | 0.41 $\boldsymbol{\pm}$ 0.11 |
|  | n | 4 |
| **Dr-sstr2a** | pEC_50_^a^ $\boldsymbol{\pm}$ S.E.M. | 7.77 $\boldsymbol{\pm}$ 0.26 |
|  | EC_50_ (nM) | 16.98 |
|  | E_max_^b^ $\boldsymbol{\pm}$ S.E.M. | 118.71 $\boldsymbol{\pm}$ 11.68 |
|  | Span $\boldsymbol{\pm}$S.E.M. | 125.90 $\boldsymbol{\pm}$ 18.64 |
|  | Hill Slope | 0.34 $\boldsymbol{\pm}$ 0.084 |
|  | n | 4 |

Values were generated when the data were fitted to the four-parameter logistic equation. Means ± S.E.M of n individual result sets were shown.

^a^ Negative logarithm of agonist concentration when reaching half maximal response.

^b^ % of maximal response observed when stimulated with ligands relative to SS-14.

Table S8. Pharmacological parameters of subfraction # 32-20 induced-hSSTR_2_, Dr-sstr2a and Dr-sstr2b activation measured by PRESTO-Tango β-arrestin recruitment assay.

|  | Test ligands | **# 32-20** |
| --- | --- | --- |
| **hSSTR_1_ ^c^** | pEC_50_^a^ $\boldsymbol{\pm}$ S.E.M. | N.D. |
|  | EC_50_ (nM) |  |
|  | E_max_^b^ $\boldsymbol{\pm}$ S.E.M. |  |
|  | Span $\boldsymbol{\pm}$S.E.M. |  |
|  | Hill Slope |  |
|  | n | 6 |
| **hSSTR_2_** | pEC_50_^a^ $\boldsymbol{\pm}$ S.E.M. | 6.52 $\boldsymbol{\pm}$ 0.03 |
|  | EC_50_ (nM) | 302.01 |
|  | E_max_^b^ $\boldsymbol{\pm}$ S.E.M. | 89.42 $\boldsymbol{\pm}$ 2.26 |
|  | Span $\boldsymbol{\pm}$S.E.M. | 88.53 $\boldsymbol{\pm}$ 1.67 |
|  | Hill Slope | 1.05 $\boldsymbol{\pm}$ 0.06 |
|  | n | 6 |
| **hSSTR_3_ ^c^** | pEC_50_^a^ $\boldsymbol{\pm}$ S.E.M. | N.D. |
|  | EC_50_ (nM) |  |
|  | E_max_^b^ $\boldsymbol{\pm}$ S.E.M. |  |
|  | Span $\boldsymbol{\pm}$S.E.M. |  |
|  | Hill Slope |  |
|  | n | 6 |
| **hSSTR_4_ ^c^** | pEC_50_^a^ $\boldsymbol{\pm}$ S.E.M. | N.D. |
|  | EC_50_ (nM) |  |
|  | E_max_^b^ $\boldsymbol{\pm}$ S.E.M. |  |
|  | Span $\boldsymbol{\pm}$S.E.M. |  |
|  | Hill Slope |  |
|  | n | 6 |
| **hSSTR_5_ ^c^** | pEC_50_^a^ $\boldsymbol{\pm}$ S.E.M. | N.D. |
|  | EC_50_ (nM) |  |
|  | E_max_^b^ $\boldsymbol{\pm}$ S.E.M. |  |
|  | Span $\boldsymbol{\pm}$S.E.M. |  |
|  | Hill Slope |  |
|  | n | 6 |
| **Dr-sstr2a** | pEC_50_^a^ $\boldsymbol{\pm}$ S.E.M. | 6.86 $\boldsymbol{\pm}$ 0.04 |
|  | EC_50_ (nM) | 138.04 |
|  | E_max_^b^ $\boldsymbol{\pm}$ S.E.M. | 87.35 $\boldsymbol{\pm}$ 1.64 |
|  | Span $\boldsymbol{\pm}$S.E.M. | 86.00 $\boldsymbol{\pm}$ 2.18 |
|  | Hill Slope | 1.30 $\boldsymbol{\pm}$ 0.13 |
|  | n | 8 |
| **Dr-sstr2b** | pEC_50_^a^ $\boldsymbol{\pm}$ S.E.M. | 7.22 $\boldsymbol{\pm}$ 0.07 |
|  | EC_50_ (nM) | 60.26 |
|  | E_max_^b^ $\boldsymbol{\pm}$ S.E.M. | 85.45 $\boldsymbol{\pm}$ 2.54 |
|  | Span $\boldsymbol{\pm}$S.E.M. | 81.88 $\boldsymbol{\pm}$ 3.65 |
|  | Hill Slope | 1.39 $\boldsymbol{\pm}$ 0.27 |
|  | n | 6 |

Values were generated when the data were fitted to the four-parameter logistic equation. Means ± S.E.M of n individual result sets were shown.

^a^ Negative logarithm of agonist concentration when reaching half maximal response.

^b^ % of maximal response observed when stimulated with ligands relative to SS-14.

^c^ Pharmacological parameters cannot be determined due to weak or inactivation of SSTRs.

Table S9. Pharmacological parameters of hSSTR_2_, Dr-sstr2a and Dr-sstr2b activation induced by des-glyco nG1 measured by PRESTO-Tango β-arrestin recruitment assay.

| **Receptor** | Test ligands | **SS-14** | #32-20 | des-glyco nG1 |
| --- | --- | --- | --- | --- |
| **hSSTR_2_** | pEC_50_^a^ $\boldsymbol{\pm}$ S.E.M. | 7.67 $\boldsymbol{\pm}$ 0.10 | 8.31 $\boldsymbol{\pm}$ 0.04 | 7.25 $\boldsymbol{\pm}$ 0.05 |
|  | EC_50_ (nM) | 21.42 | 48.99 | 56.35 |
|  | E_max_^b^ $\boldsymbol{\pm}$ S.E.M. | 101.4 $\boldsymbol{\pm}$ 4.37 | 79.03 $\boldsymbol{\pm}$ 1.52 | 97.11 $\boldsymbol{\pm}$ 2.65 |
|  | Span $\boldsymbol{\pm}$S.E.M. | 98.79 $\boldsymbol{\pm}$ 5.77 | 78.86 $\boldsymbol{\pm}$ 1.96 | 97.20 $\boldsymbol{\pm}$ 3.16 |
|  | Hill Slope | 0.69 $\boldsymbol{\pm}$ 0.10 | 0.98 $\boldsymbol{\pm}$ 0.08 | 1.09 $\boldsymbol{\pm}$ 0.11 |
|  | n | 6 | 4 | 6 |
| **Dr-sstr2a** | pEC_50_^a^ $\boldsymbol{\pm}$ S.E.M. | 6.76 $\boldsymbol{\pm}$ 0.14 | 8.74 $\boldsymbol{\pm}$ 0.05 | 6.53 $\boldsymbol{\pm}$ 0.09 |
|  | EC_50_ (nM) | 173.3 | 1.82 | 296.4 |
|  | E_max_^b^ $\boldsymbol{\pm}$ S.E.M. | 109.39 $\boldsymbol{\pm}$ 9.41 | 82.35 $\boldsymbol{\pm}$ 1.92 | 68.55 $\boldsymbol{\pm}$ 4.56 |
|  | Span $\boldsymbol{\pm}$S.E.M. | 109.0 $\boldsymbol{\pm}$ 10.32 | 80.82 $\boldsymbol{\pm}$ 2.74 | 65.90 $\boldsymbol{\pm}$ 4.87 |
|  | Hill Slope | 0.72 $\boldsymbol{\pm}$ 0.12 | 1.18 $\boldsymbol{\pm}$ 0.15 | 0.95 $\boldsymbol{\pm}$ 0.13 |
|  | n | 6 | 4 | 6 |
| **Dr-sstr2b** | pEC_50_^a^ $\boldsymbol{\pm}$ S.E.M. | 7.75 $\boldsymbol{\pm}$ 0.12 | 9.27 $\boldsymbol{\pm}$ 0.24 | 8.71 $\boldsymbol{\pm}$ 0.14 |
|  | EC_50_ (nM) | 17.6 | 0.54 | 1.97 |
|  | E_max_^b^ $\boldsymbol{\pm}$ S.E.M. | 107.49 $\boldsymbol{\pm}$ 5.82 | 89.80 $\boldsymbol{\pm}$ 6.43 | 92.57 $\boldsymbol{\pm}$ 3.75 |
|  | Span $\boldsymbol{\pm}$S.E.M. | 105.84 $\boldsymbol{\pm}$ 8.30 | 88.23 $\boldsymbol{\pm}$ 13.37 | 96.18 $\boldsymbol{\pm}$ 8.60 |
|  | Hill Slope | 0.47 $\boldsymbol{\pm}$ 0.07 | 0.54 $\boldsymbol{\pm}$ 0.17 | 0.53 $\boldsymbol{\pm}$ 0.10 |
|  | n | 6 | 3 | 6 |

Values were generated when the data were fitted to the four-parameter logistic equation. Means ± S.E.M of n individual result sets were shown.

^a^ Negative logarithm of agonist concentration when reaching half maximal response.

^b^ % of maximal response observed when stimulated with ligands relative to SS-14.

**Reference**

1 Masuho, I., Martemyanov, K. A. & Lambert, N. A. Monitoring G Protein Activation in Cells with BRET. *Methods Mol Biol* **1335**, 107-113, doi:10.1007/978-1-4939-2914-6_8 (2015).
