# Supplementary material for: Fish-hunting cone snail disrupts prey’s glucose homeostasis with weaponized mimetics of somatostatin and insulin
Source: Nat Commun. 2024 Aug 20;15:6408. doi: 10.1038/s41467-024-50470-2 (PMC11336141; doi:10.1038/s41467-024-50470-2)
Supplement: Supplementary file 3 — Reporting Summary [file 41467_2024_50470_MOESM3_ESM.pdf]

nature portfolio

Corresponding author(s): Helena Safavi-Hemami
Last updated by author(s): Jun 14, 2024

Reporting Summary

Nature Portfolio wishes to improve the reproducibility of the work that we publish. This form provides structure for consistency and transparency in reporting. For further information on Nature Portfolio policies, see our Editorial Policies and the Editorial Policy Checklist.

Statistics

For all statistical analyses, confirm that the following items are present in the figure legend, table legend, main text, or Methods section.

n/a	Confirmed	
☐	☒	The exact sample size (*n*) for each experimental group/condition, given as a discrete number and unit of measurement
☐	☒	A statement on whether measurements were taken from distinct samples or whether the same sample was measured repeatedly
☐	☒	The statistical test(s) used AND whether they are one- or two-sided<br>*Only common tests should be described solely by name; describe more complex techniques in the Methods section.*
☐	☒	A description of all covariates tested
☐	☒	A description of any assumptions or corrections, such as tests of normality and adjustment for multiple comparisons
☐	☒	A full description of the statistical parameters including central tendency (e.g. means) or other basic estimates (e.g. regression coefficient) AND variation (e.g. standard deviation) or associated estimates of uncertainty (e.g. confidence intervals)
☐	☒	For null hypothesis testing, the test statistic (e.g. *F*, *t*, *r*) with confidence intervals, effect sizes, degrees of freedom and *P* value noted<br>*Give P values as exact values whenever suitable.*
☒	☐	For Bayesian analysis, information on the choice of priors and Markov chain Monte Carlo settings
☒	☐	For hierarchical and complex designs, identification of the appropriate level for tests and full reporting of outcomes
☒	☐	Estimates of effect sizes (e.g. Cohen's *d*, Pearson's *r*), indicating how they were calculated

Our web collection on statistics for biologists contains articles on many of the points above.

Software and code

Policy information about availability of computer code

Data collection	not applicable
Data analysis	Graphpad Prism (v.9.4.1), fqtrim (v0.9.4), Trinity (v2.2.1), mafft (v7.520), IQ-tree (v2.2.2.3), exonerate (v2.76.2), Byonic software (v2.16 Protein Metrics), BioPharma Finder Software (Thermo Fisher Scientific), Preoteome Discoverer 2.5.0.400 (Thermo Fisher Scientific Inc.), Skyline 22.2.0.351 (ProteoWizard)

For manuscripts utilizing custom algorithms or software that are central to the research but not yet described in published literature, software must be made available to editors and reviewers. We strongly encourage code deposition in a community repository (e.g. GitHub). See the Nature Portfolio guidelines for submitting code & software for further information.

Data

Policy information about availability of data

All manuscripts must include a data availability statement. This statement should provide the following information, where applicable:

- Accession codes, unique identifiers, or web links for publicly available datasets
- A description of any restrictions on data availability
- For clinical datasets or third party data, please ensure that the statement adheres to our policy

The sequencing data generated in this study have been deposited in the NCBI Short Sequence Archive (SRA) under the following accession number: SRR27535905 Section B4, SRR27535906 Section B3, SRR27535907 Section B2, SRR27535908 Section B1. The mass spectrometry proteomics data have been deposited to the ProteomeXchange Consortium via the PRIDE [1] partner repository with the dataset identifier PXD053107 and 10.6019/PXD053107.

Research involving human participants, their data, or biological material

Policy information about studies with human participants or human data. See also policy information about sex, gender (identity/presentation), and sexual orientation and race, ethnicity and racism.

Reporting on sex and gender	N/A
Reporting on race, ethnicity, or other socially relevant groupings	N/A
Population characteristics	N/A
Recruitment	N/A
Ethics oversight	N/A

Note that full information on the approval of the study protocol must also be provided in the manuscript.

Field-specific reporting

Please select the one below that is the best fit for your research. If you are not sure, read the appropriate sections before making your selection.

☒ Life sciences ☐ Behavioural & social sciences ☐ Ecological, evolutionary & environmental sciences

For a reference copy of the document with all sections, see nature.com/documents/nr-reporting-summary-flat.pdf

Life sciences study design

All studies must disclose on these points even when the disclosure is negative.

Sample size	No sample size calculations were performed. All experiments were performed using at least 3 biological replicates and 2 technical replicates.
Data exclusions	Pancreases not exhibiting robust glucagon secretion at low glucose levels were excluded from the data analysis.
Replication	information on all biological and experimental replicates are provided for each experiment in the manuscript or in the supporting information.
Randomization	not applicable
Blinding	Wherever possible, experiments using pancreas perfusion were done under blinded conditions.

Reporting for specific materials, systems and methods

We require information from authors about some types of materials, experimental systems and methods used in many studies. Here, indicate whether each material, system or method listed is relevant to your study. If you are not sure if a list item applies to your research, read the appropriate section before selecting a response.

Materials & experimental systems

n/a	Involved in the study	
	x	Antibodies
	x	Eukaryotic cell lines
x		Palaeontology and archaeology
	x	Animals and other organisms
x		Clinical data
x		Dual use research of concern
x		Plants

Methods

n/a	Involved in the study	
x		ChIP-seq
x		Flow cytometry
x		MRI-based neuroimaging

Antibodies

Antibodies used	Glucagon concentration in perfusion samples were measured by a validated in-house RIAs using the 4305 antibody, which reacts with the C-terminal epitope of glucagon and no other cleavage products of pro-glucagon. Insulin concentrations were measured with the 2006 antibody, which measures all isoforms of rat insulin. Somatostatin concentrations were measured using the 1758 antibody, which detects both SS-14 and SS-28 (Xu, S. F. S., Andersen, D. B., Izarzugaza, J. M. G., Kuhre, R. E. & Holst, J. J. Acta Physiol (Oxf) 229, e13464, doi:10.1111/apha.13464 (2020)).
Validation	The antibody used in this study were validated in a previous study: Xu, S. F. S., Andersen, D. B., Izarzugaza, J. M. G., Kuhre, R. E. & Holst, J. J. Acta Physiol (Oxf) 229, e13464, doi:10.1111/apha.13464 (2020).

Eukaryotic cell lines

Policy information about cell lines and Sex and Gender in Research

Cell line source(s)	HTLA cells were given by Prof. Bryan Roth (University of North Carolina, Chapel Hill, USA) and HEK293T cells were given by Prof. Hans Braüner-Osborne (University of Copenhagen, Denmark).
Authentication	Cells were not authenticated prior to use.
Mycoplasma contamination	The cell lines were not routinely tested for mycoplasma contamination during this study.
Commonly misidentified lines (See ICLAC register)	not applicable

Animals and other research organisms

Policy information about studies involving animals; ARRIVE guidelines recommended for reporting animal research, and Sex and Gender in Research

Laboratory animals	Adult male Wistar rats and C57BL/6J female mice (12-20 weeks)
Wild animals	Venom was extracted from the cone snail Conus geographus collected from the wild and typically sacrificed within 24 hours following collection.
Reporting on sex	Male rats and female mice were used.
Field-collected samples	The cone snail Conus geographus was collected from the wild, kept in seawater (room temperature) following collection and typically sacrificed within 24 hours following collection.
Ethics oversight	Animal experiments were conducted with permission from the Danish Animal Experiments Inspectorate (License Number 2018-15-0201-01397) in according to guidelines set by Danish legislation and the European Union Directive.

Note that full information on the approval of the study protocol must also be provided in the manuscript.

Plants

Seed stocks	N/A
Novel plant genotypes	N/A
Authentication	N/A
